# Antenatal dexamethasone use and respiratory distress in late preterm infants: results from first Vietnamese matched cohort study

**DOI:** 10.1186/s12884-021-04019-6

**Published:** 2021-08-07

**Authors:** Tran Tuan Hung Ho, Quang Vinh Truong, Thi Kim Anh Nguyen, Minh Tam Le, Vu Quoc Huy Nguyen

**Affiliations:** Department of Obstetrics & Gynecology, Hue University of Medicine and Pharmacy, Hue University, Hue, 49120 Vietnam

**Keywords:** Late preterm infant, Antenatal dexamethasone, Respiratory distress

## Abstract

**Background:**

Respiratory distress syndrome (RDS) is one of the leading causes of early neonatal morbidity and mortality in late preterm infants (LPIs) worldwide. This matched cohort study aimed to assess how the antenatal dexamethasone use affect the respiratory distress (RD) proportion in preterm newborns between 34 0/7 weeks and 36 6/7 weeks of gestation.

**Methods:**

This was a prospective cohort study on 78 women with singleton pregnancy who were in threatened preterm birth and had not received prior dexamethasone, who were admitted between 34 0/7 weeks and 36 6/7 weeks at Hue University of Medicine and Pharmacy Hospital from June 2018 to May 2020. The matched control group without dexamethasone use included 78 pregnant women diagnosed with threatened late preterm births who were at similar gestational ages and estimated fetal weights as the treatment group. The treatment group received 6 mg intramuscular dexamethasone every 12 h for a total of 4 doses or until delivery. Primary outcome was the rate of neonatal RD. Secondary neonatal outcomes included the need for respiratory support, neonatal intensive care unit (NICU) admission, hypoglycemia, necrotizing enterocolitis, intraventricular hemorrhage, and neonatal death. Statistical analyses were performed by using SPSS software, version 26.0.

**Results:**

The proportion of RD in LPI was significantly lower in the treatment group than in the matched control group (10.3% vs. 23.1%, respectively), adjusted Odds Ratio [aOR] 0.29; 95% confidence interval [CI] 0.10 – 0.83 and p = 0.021. Neonatal hypoglycemia was more common in the dexamethasone group than in the matched group (25.6% vs. 12.8%, respectively; aOR, 2.59; 95% CI, 1.06 – 6.33; p = 0.037). There were no significant between-groups differences in the incidence of respiratory support, NICU admission or length of hospital stay.

**Conclusions:**

Administration of antenatal dexamethasone to women at risk for late preterm birth could help to lower the proportion of respiratory distress in late preterm infants.

**Supplementary Information:**

The online version contains supplementary material available at 10.1186/s12884-021-04019-6.

## Background

Preterm birth is defined as alive birth before the completion of 37 weeks of pregnancy [1]. Preterm infants are at higher risk of mortality and morbidity, including long-term issues affecting quality of life. According to the World Health Organization, 15 million preterm babies are born annually, and the rate of preterm birth is rising [1]. Infants who are born from 34 weeks – 36 weeks and 6 days, also known as late preterm infants, are at higher risks of respiratory distress and other complications than those born at term [2]. In 2010, the rate of preterm birth from 32 to 37 weeks was approximately 80% in Southeast Asia in general, particularly in Vietnam [1]. Respiratory distress syndrome (RDS) is one of the leading causes of early neonatal morbidity and mortality. Although the incidence of RDS is less common after 34 weeks of gestation, late preterm newborns are more likely to experience RDS and other neonatal respiratory complications than infants born at term [3].

Prophylactic corticosteroids in singleton preterm pregnancies accelerate lung maturation and reduce the rate of RDS. There is strong evidence that corticosteroids can reduce the risk of neonatal respiratory complications when administered before 34 weeks of gestation [4, 5]. Administration of antenatal corticosteroids (ACS) has become more common following a consensus from the National Institutes of Health (NIH) in 1994 [6]. Both betamethasone and dexamethasone are effective in accelerating fetal lung maturation and are the most widely approved for use in ACS therapy [7]. However, evidence for the benefit of ACS in preterm pregnancy between 34 weeks 0 day and 36 weeks 6 days is controversial due to a limitation of available data. To clarify this issue, the Antenatal Late Preterm Steroids (ALPS) trial was conducted [8]. This was a multicenter, randomized controlled trial that assessed the benefits of betamethasone administration for women with singleton gestation pregnancies at risk for late preterm birth. The ALPS trial findings were published in early 2016 and showed a significant reduction in neonatal respiratory complications in response to betamethasone administration. With the publication of ALPS trial, the use of antenatal corticosteroids in this population has become increasingly common in clinical practice for the management of pregnancies at risk for late preterm birth [9]. Recently, the American College of Obstetricians and Gynecologists recommended a single course of corticosteroids for pregnant women at risk of late preterm delivery within 7 days who have not received a previous course of antenatal corticosteroids if proceeding with induction or delivery in no less than 24 h and no more than 7 days [10].

In Vietnam, ACS was recommended for women at risk of preterm birth between 26 and 34 weeks of gestation, and corticosteroids’ coverage was only 52% [11]. However, medical literature searches in Vietnam through the end of 2017 retrieved no work on the use of ACS in the late preterm period, and current national guidelines on reproductive health services have not covered this issue. Furthermore, neonatal care interventions for late preterm newborns are considerably limited in low-resource countries. This study aimed to assess how the antenatal dexamethasone use affect the respiratory distress proportion in premature newborns between 34 0/7 weeks and 36 6/7 weeks of gestation.

## Methods

This prospective matched cohort study has been carried out at the Department of Obstetrics & Gynecology of the Hue University of Medicine and Pharmacy Hospital between June 2018 and May 2020.

Non-probability comprehensive sampling method was conducted. Sample size was calculated by using the following formula:

$$n=\frac{{Z}_{1-\alpha /2}^{2}P(1-P)}{{d}^{2}}$$

where n was the sample size, Z was the statistic corresponding to level of confidence with $\alpha$ = 95% (Z = 1.96), P was the expected prevalence of respiratory distress syndrome of late preterm newborns who were exposed to antenatal corticosteroid (P = 5.2%, based on Attawattanakul et al. [12]), and d = 0.05. The required minimum number of study population in each group was 76 subjects.

**Fig. 1:**
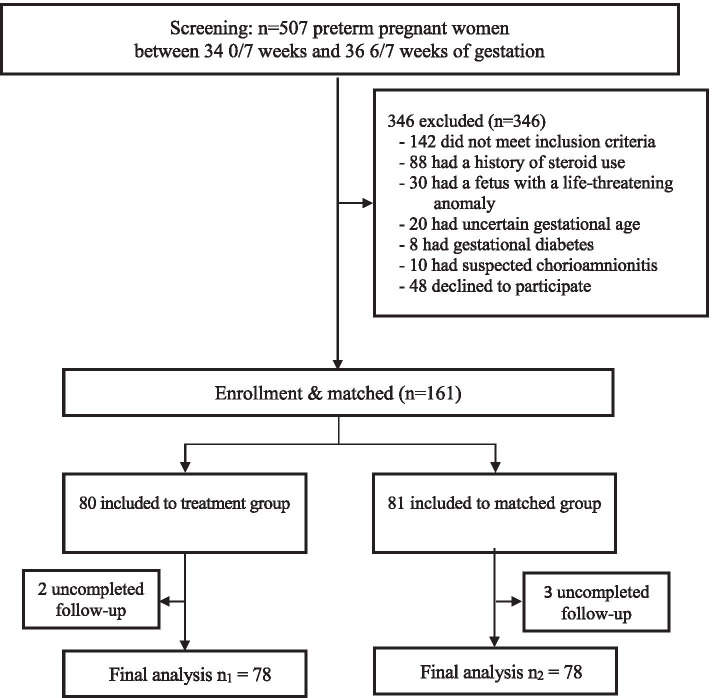
Study participants flowchart

Study subjects included 78 pregnant women with singleton pregnancies from 34 0/7 weeks to 36 6/7 weeks, estimated based on the first day of the last menstrual period or the estimated delivery date of first trimester ultrasound, having one or more of the following signs: 1) spontaneous rupture of the membranes; 2) preterm labor, defined as having 4 contractions in 20 min or 8 contractions in 60 min causing cervical dilation, or cervical dilation ≥ 2 cm in the presence of contractions; and 3) high probability of indication for late preterm delivery: uncontrolled bleeding placenta previa, placental abruption, or elective cesarean section due to maternal medical conditions. Exclusion criteria were previous administration of dexamethasone during pregnancy, stillbirth and life-threatening fetal anomalies, gestational diabetes, chorioamnionitis, or contraindications for dexamethasone. The treatment group received dexamethasone 6 mg intramuscularly every 12 h for a total of 4 doses or until delivery. The matched control group included 78 newborn infants who were at similar gestational age at delivery with similar estimated fetal weight to the treatment group born to mothers who had not received dexamethasone before delivery. Matching was done by using range of gestational age from 34 weeks 0 day to 36 weeks 6 days and estimated fetal weight from 1600 to 3000 g. Study participants enrollment and follow-up is presented in Fig. 1.

Maternal data collection was performed by interviewing and examining pregnant women with respect to maternal age, gravidity, history of preterm delivery, and gestational age. Follow-up was performed during the treatment course to collect data on the cause of pregnancy termination, mode of delivery, other maternal outcomes; birthweight and neonatal outcomes (see Supplement, “Data collection tool”).

Pregnant women in preterm labor were given tocolytic agent (nifedipine) in accordance with the institutional recommended practice. If the woman delivered baby before receiving full dose of dexamethasone, she was still analyzed in the treatment group.

Late preterm newborns were evaluated after birth to collect the following outcome information:

- The primary outcome was the rate of respiratory distress within 72 h after birth, diagnosed by the presence of one or more of the following criteria: 1) dyspnea (respiratory rate ≥ 60/minute or < 30/minute, apnea ≥ 20 s); 2) central cyanosis (blue tongue and lips); 3) chest indrawing and 4) grunting on expiration. Classification of respiratory distress includes mild, moderate and severe levels based on respiratory rate and present/ absent grunting or chest indrawing [13].

- Neonatal secondary outcomes included the following: respiratory support (supplemental oxygen through nasal cannula, the use of continuous positive airway pressure (CPAP) or mechanical ventilation), surfactant administration, respiratory distress syndrome (defined as the presence of clinical signs of respiratory distress, rapid onset of severe hypoxemia on blood gas analysis and abnormal chest radiography showing a ground-glass reticulo-granular appearance with air bronchograms; radiological appearances correlating with clinical severity and classified as mild, moderate and severe neonatal respiratory distress syndrome) [14, 15], Apgar’s score < 7 at 1 min and 5 min after birth, need for resuscitation at birth, neonatal intensive care unit (NICU) admission and length of hospital stay; maximal follow-up time of first 28 days of life.

- Other neonatal outcomes included the following: early-onset neonatal sepsis, jaundice requiring phototherapy, hypoglycemia (defined as a glucose level less than 2.2 mmol/L), intraventricular hemorrhage, necrotizing enterocolitis (defined as modified Bell’s Staging Criteria including stage I – suspected, stage II – definite and stage III – advanced), and neonatal death before discharge (within the first 28 days of life).

Maternal outcomes included chorioamnionitis (maternal fever > 37.8 °C, maternal tachycardia > 100/min, fetal tachycardia > 160/min, foul smelling amniotic fluid, white blood cell count > 15,000, C-reactive protein > 20 mg/L), endometritis (maternal fever > 37.8 °C, maternal tachycardia > 100/min, uterine tenderness, foul smelling lochia), retained placenta and the length of hospital stay after delivery.

Data were collected and analyzed using Statistical Package for the Social Sciences (SPSS) software, version 26.0. Continuous variables are expressed as the mean ± standard deviation or median (interquartile range – IQR) and were compared using t-tests (for normal distribution) or Mann–Whitney tests (for non-normal distribution). Categorical variables were compared with Chi-square and Fisher’s exact tests. Relative risks (RRs) and 95% confidence intervals (CIs) are reported. Multivariable logistic or Poisson regression model was conducted. The association between the outcome and categorical variables were estimated as Odds Ratios (ORs) adjusted for membrane rupture and preterm labor, reported with 95% confidence intervals. Since the sample size was not very large, we performed comparison of the entire group and not in pair-wise approach. P-value of < 0.05 was considered statistically significant.

The study protocol has been approved by the Ethics Committee for Biomedical Research of Hue University of Medicine and Pharmacy, approval number H2018/357. Written consent was obtained from all study participants before their enrollment.

## Results

There were no significant differences between dexamethasone group and matched group with respect to maternal age, gravidity, fetal weight, history of prematurity, or method of delivery. The most frequent cause of preterm birth was preterm premature rupture of membranes (43.6%) (Table 1).

**Table 1:** General characteristics of the study subjects

Characteristics	Dexamethasone group (n_1_ = 78)	Matched group (n_2_ = 78)	p-value
Maternal age (year) Mean (± SD)	28.5 ± 5.1	27.6 ± 6.0	0.35
Gravidity, n (%)			
1	35 (44.9)	33 (42.3)	0.75
≥ 2	43 (55.1)	45 (57.7)	
History of preterm birth, n (%)	8 (10.3)	7 (9.0)	0.79
Gestational age at birth, n (%)			
34^0/7^ – 34^6/7^	6 (7.7)	5 (6.4)	0.75
35^0/7^ – 35^6/7^	31 (39.7)	31 (39.7)	1.00
36^0/7^ – 36^6/7^	41 (52.6)	42 (53.8)	0.87
Birth weight, (gram) Mean (± SD)	2547 ± 507	2488 ± 344	0.40
Causes of preterm birth, n (%)			
Rupture of membranes	21 (26.9)	46 (59.0)	< 0.0001
Preterm labor	41 (52.6)	27 (34.6)	0.02
Preeclampsia	6 (7.7)	1 (1.3)	0.12
Previa placenta	3 (3.8)	0	-
Placental abruption	0	1 (1.3)	-
Fetal compromise	6 (7.7)	2 (2.6)	0.28
Elective	1 (1.3)	0	-
Mode of delivery, n (%)			
Normal delivery	48 (61.5)	53 (67.9)	0.40
Cesarean delivery	30 (38.5)	22 (28.2)	0.17
Operative delivery (forceps)	0	3 (3.8)	-

The percentage of respiratory distress was significantly lower in the dexamethasone group than in the matched control group (10.3% vs. 23.1%, respectively; aOR, 0.29; 95% CI, 0.10 – 0.83; p = 0.021) (Table 2). There was a difference in the level of breathing difficulty related to respiratory distress in late preterm infants between the two groups. In particular, there were 2 infants with moderate levels in the dexamethasone group and 10 infants with moderate levels in the matched group.

**Table 2:** Neonatal respiratory outcomes and related conditions between the dexamethasone and matched control groups

**Outcomes**	**Dexamethasone group** **(n**_**1**_** = 78)**	**Matched** **group** **(n**_**2**_** = 78)**	**Unadjusted** **OR (95% CI);** **p**	**Adjusted** ^a^ **OR (95% CI);** **p**
Respiratory distress, n (%)	8 (10.3)	18 (23.1)	0.38 (0.16–0.94); 0.036	0.29 (0.10–0.83); 0.021
Mild	5 (6.4)	7 (9.0)	0.70 (0.21–2.29);	0.53 (0.14–2.01); 0.349
Moderate	2 (2.6)	10 (12.8)	0.550	0.18 (0.35–0.98); 0.047
Severe	(1.3)	1 (1.3)	0.18 (0.04–0.85);	0.44 (0.03–7.86); 0.577
			0.030	
			1.00 (0.06–16.28); 1.00	
Respiratory support, n (%)				
Supplemental oxygen	15 (19.2)	19 (24.4)	0.74 (0.34–1.59); 0.444	0.71 (0.31–1.66); 0.431
CPAP	2 (2.6)	5 (6.4)	0.38 (0.07–2.04); 0.262	0.22 (0.03–1.53); 0.127
Mechanical ventilation	1 (1.3)	1 (1.3)	1.00 (0.06–16.28);	0.44 (0.03–7.86);
			1.00	0.577
Surfactant use, n (%)	1 (1.3)	1 (1.3)	1.00 (0.06–16.28); 1.00	0.44 (0.03–7.86); 0.577
Resuscitation at birth, n (%)	10 (12.8)	11 (14.1)	0.90 (0.36–2.25); 0.815	0.72 (0.26–2.05); 0.541
Apgar score, n (%)				
< 7 at 1 min	2 (2.6)	2 (2.6)	1.00 (00.14–7.28);	0.58 (0.07–5.12);
< 7 at 5 min	1 (1.3)	0	1.00	0.620
			-	-
Respiratory distress syndrome, n (%)	1 (1.3)	1 (1.3)	1.00 (0.06–16.28); 1.00	0.44 (0.03–7.86); 0.577
NICU admission, n (%)	24 (30.8)	32 (41.0)	0.64 (0.33–1.24); 0.183	0.57 (0.28–1.17); 0.570
Median length of hospital stay (IQR), days^b^	5 (3.0 – 6.0)	5 (3.0 – 6.25)	1.01^b^ (0.88–1.16); 0.863	0.96^b^ (0.83–1.12); 0.600

^a^Multivariate analysis was implemented for adjustment the effects of the causes of preterm birth by logistic regression model or Poisson regression model

^b^the Poisson regression model; IRR with 95% confidence interval was used to evaluate the magnitude of hospital stay between two groups

The rate of respiratory distress syndrome, Apgar score < 7, need for resuscitation at birth, NICU admission, surfactant use, and length of hospital stay were not significantly different between the two groups. Apgar score ≥ 7 at 1 min and 5 min after birth accounted for the majority in the two groups.

There were no cases of intraventricular hemorrhage, necrotizing enterocolitis, or neonatal death in the two groups, as shown in Table 3. Compared to the matched control group, dexamethasone use did not significantly reduce the incidence of early-onset neonatal sepsis or jaundice requiring phototherapy (p > 0.05). The percentage of hypoglycemia was significantly higher in the dexamethasone group than in the matched group (25.6% vs. 12.8%; aOR, 2.59; 95% CI, 1.06 – 6.33; p = 0.037). Two cases with severe breathing difficulty in the two groups were due to respiratory distress syndrome (stage III) and were treated with mechanical ventilation and surfactant therapy.

**Table 3:** Other neonatal secondary outcomes

**Outcomes,** **n (%)**	**Dexamethasone group** **(n**_**1**_** = 78)**	**Matched** **group** **(n**_**2**_ ** = 78)**	**Unadjusted** **OR (95% CI); p**	**Adjusted**^a^ **OR (95% CI); p**
Early-onset neonatal sepsis	16 (20.5)	22 (28.2)	0.66 (0.31—1.38);0.265	0.51 (0.23–1.14); 0.100
Jaundice requiring phototherapy	12 (15.4)	17 (21.8)	0.65 (0.29–1.48); 0.305	0.67 (0.28–1.60); 0.365
Hypoglycemia	20 (25.6)	10 (12.8)	2.35 (1.02–5.41);0.046	2.59 (1.06–6.33); 0.037
Intraventricular hemorrhage	0	0	-	-
Necrotizing enterocolitis	0	0	-	-
Neonatal death	0	0	-	-

^a^Multivariate analysis was implemented for adjustment the effects of the causes of preterm birth by logistic regression model or Poisson regression model

Regarding maternal outcomes, as shown in Table 4, there were no significant between-group differences in the incidence of endometritis, chorioamnionitis or retained placenta. There was only 1 case of chorioamnionitis in the matched group, and there were no diagnosed endometritis cases in either group. The median postpartum length of hospital stay in the dexamethasone group was slightly higher than in the matched group, but the difference was not statistically significant (p > 0.05).

**Table 4:** Maternal outcomes

**Outcome**	**Dexamethasone group** **(n**_**1**_** = 78)**	**Matched** **group** **(n**_**2**_** = 78)**	**Unadjusted** **OR (95% CI); p**	**Adjusted**^a^ **OR (95% CI); p**
Chorioamnionitis, n (%)	0	1 (1.3)	-	-
Endometritis, n (%)	0	0	-	-
Retained placenta, n (%)	9 (11.5)	7 (9.0)	1.32 (0.47–3.75); 0.599	2.04 (0.67–6.20); 0.210
Median postpartum length of hospital stay (IQR), days	5 (3.0 – 5.0)	4 (3.0 – 5.0)	1.04^b^ (0.90–1.20); 0.604	0.95^b^(0.82–1.11); 0.549

^a^Multivariate analysis was implemented for adjustment the effects of the causes of preterm birth by logistic regression model or Poisson regression model

^b^the Poisson regression model; IRR with 95% confidence interval was used to evaluate the magnitude of postpartum length between two groups

## Discussion

In this matched cohort study from Vietnam, which consists of 78 cases in each dexamethasone group and matched control group, we found that dexamethasone administration to women at risk at 34 0/7 weeks—36 6/7 weeks of gestation could help to lower the proportion of respiratory distress among their newborns (aOR 0.29; 95%CI: 0.10 – 0.83, p = 0.021).

To date, many studies around the world have been conducted to evaluate the health and economic effects of ACS administered to a special population of late preterm infants. The antenatal corticosteroids use among pregnant women between 34 and 36 weeks 6 days was accepted as an effective approach that can significantly reduce the cost and acute morbidity associated with late preterm birth [16]. The mechanism that causes respiratory distress in babies born between 22 and 34 weeks of gestation, including surfactant deficiency and immature development of the fetal lung, can also affect late preterm infants. Delayed opening of the epithelial sodium channel responsible for clearance of the fluid and deficiency of pulmonary surfactant plays a key role in the pathophysiology of respiratory morbidity [2]. Antenatal corticosteroids may have an impact on both surfactant maturity and fetal lung fluid clearance in late preterm newborns [17]. Currently, Vietnam is a lower middle-income country, but preterm newborn care is still limited. The economic costs of preterm birth are important in terms of neonatal intensive care and long-term complications [1]. Therefore, a low-cost intervention, such as antenatal dexamethasone, may indeed help to alleviate the neonatal interventions needed in Vietnam.

Previously, Yinon et al. (2012) conducted a retrospective cohort study to compare the outcomes of infants born between 34 and 37 weeks of gestation who had either received betamethasone or not. The rate of the composite respiratory morbidity outcome was significantly higher in the nontreatment group than in the betamethasone group (21% vs. 8.4%, respectively; p = 0.02). However, this was a retrospective cohort study design, and the authors could not control for potential confounding factors [18]. According to Attawattanakul (2015), dexamethasone administration in late preterm labor significantly decreased the rate of respiratory distress without increasing the rate of adverse events. Furthermore, only 6% of participants completed the full course of antenatal dexamethasone [12]. Most recently, both Gyamfi-Bannerman et al. (2016) and Uquillas et al. (2020) found that antenatal betamethasone decreased the need for substantial respiratory support during the first 72 h of late preterm newborns after birth (p = 0.02) [8, 19]. Uquillas et al.’s study was also a retrospective cohort study, so they could not control for differences in subgroup gestational age (34 weeks, 35 weeks and 36 weeks of gestation). Despite being the largest trial, it is important to note that only 60% of the study group completed the full course of betamethasone in the Gyamfi-Bannerman et al.’s trial.

In addition, there are other studies that reported opposite results. A randomized controlled trial conducted by Porto et al. showed that the rate of respiratory morbidity was similar between betamethasone and placebo groups (25% vs. 23%, respectively). They concluded that antenatal treatment with corticosteroids was not effective in reducing neonatal respiratory morbidity. However, 43 pregnant women (13%) were discharged and lost to follow-up. [20]. Recently, Balreldin et al. (2020) even concluded that the rate of respiratory distress in the group using ACS was higher than in the group without ACS (6% vs. 4.7%, respectively) [21]. These differences may be due to the different sample sizes and trial designs; Balreldin et al. pointed out that the weakness in their study was the limited sample size; therefore, their analysis had inadequate power to identify differences. In addition, our study is the first one in Vietnam to use dexamethasone to determine the benefits of antenatal corticosteroids on respiratory distress reduction in late preterm infants.

In our matched cohort study, there were no significant differences between groups in baseline characteristics, including maternal age, history of prematurity, gravidity, and method of delivery. The most frequent cause of delivery was premature rupture of the membranes (43.6%), followed by preterm labor (42.9%). Compared to the matched group, the percentage of membrane rupture in the dexamethasone group was lower (p < 0.0001), but the percentage of preterm labor was higher (p = 0.02). These differences may be explained by the longer the rupture of membranes, the more limited the indication of dexamethasone to be used. In Viet Nam, according to the National Standards on Reproductive Healthcare, dexamethasone was less often given for ruptured membranes. There were reported risk factors for chorioamnionitis and one of the reasons was longer duration of membrane rupture. In this study, the signs of infection (maternal fever, foul smelling amniotic fluid, white blood cell count…) were closely monitored while using dexamethasone among ruptured membranes cases.

After adjusting for membrane rupture and preterm labor, neonatal respiratory distress was still significantly different between the two groups. As mentioned above, dexamethasone primarily reduced the moderate level of breathing difficulty. Although the difference was not statistically significant, antenatal dexamethasone may help improve the level of breathing difficulty in late preterm newborns.

Our study showed that there was a significant reduction in the rate of respiratory distress. Nevertheless, the rate of NICU admission, the need for respiratory support, respiratory distress syndrome, surfactant use, and Apgar score < 7 were not significantly different between the dexamethasone and matched control groups. These findings may contradict to previous Gyamfi-Bannerman et al.’ study, the effect may not much enough to decrease the percentages of need for respiratory support and surfactant use [8]. These differences may be due to the different sample sizes and NICU admission. We found that there were cases of neonatal respiratory distress at different levels. Although good resuscitation at birth significantly improved the Apgar score, some infants showed signs of respiratory distress within 72 h after birth (tachypnea, grunting, chest indrawing, etc.) and the need for respiratory support. Table 2 also shows that dexamethasone did not shorten the length of hospital stay for infants. This could be explained by many other factors affecting the length of hospital stay of late preterm newborns, including gestational age, birth weight, sex, method of delivery and neonatal complications [22].

Furthermore, administration of dexamethasone did not significantly improve the rates of short-term morbidity or complications of late preterm infants, consisting of jaundice requiring phototherapy, early-onset neonatal sepsis, intraventricular hemorrhage, necrotizing enterocolitis and neonatal death. Notably, the rate of neonatal hypoglycemia was higher in the dexamethasone group than in the matched group. Few trials of antenatal corticosteroids have recorded the same result on neonatal hypoglycemia in the late preterm period. According to Ramadan et al. (2016), infants in the betamethasone group had a higher incidence of neonatal hypoglycemia (p = 0.04) [23]. Gyamfi-Bannerman (2016) also demonstrated that betamethasone increased the rate of neonatal hypoglycemia (24.0% vs. 15.0%, respectively; RR, 1.60; 95% CI, 1.60 – 1.87; p < 0.001), although there were no reported adverse events related to neonatal hypoglycemia, and the condition was self-limiting [8]. Our findings are consistent with the results of Gyamfi-Bannerman. In late preterm period, cord serum C-peptide levels of betamethasone-exposed fetuses were higher than those of the non-exposed fetuses [24]. In addition to C-peptide levels, the cord serum glucose levels were also higher. These cause hyperinsulinemia which increase the risk of neonatal hypoglycemia in late preterm newborns after cutting the umbilical cord. Another possible explanation is that antenatal corticosteroids also may directly affect alpha and beta cells of pancreas that control glycogenolysis and gluconeogenesis associated neonatal hypoglycemia [19]. Uquillas et al.’s study found that neonatal hypoglycemia was more severe in betamethasone-treated group with the significantly lower mean initial glucose and mean glucose nadir [19]. Severe hypoglycemia may cause brain injury in late preterm infants. In our study, we routinely monitored blood glucose of newborns for the first 24 h of life in the late preterm period who were exposed to antenatal dexamethasone, and we managed hypoglycemia cases as the recommended protocol [25]. According to Neonatal Adverse Event Severity Scale (NAESS), assessing the severity of adverse events of neonatal hypoglycemia were classified as Grade 1—Mild, Grade 2—Moderate, Grade 3—Severe, Grade 4—Life threatening, and Grade 5—Death [26]. However, we do not have enough data to provide the treatment corresponding to the severity in this study.

The results in Table 4 show that dexamethasone administration did not increase the risk of infection for the mother. These findings are consistent with the results of Gyamfi-Bannerman et al. [8]. The diagnosis of maternal chorioamnionitis was based on clinical signs and confirmed by the evidence of pathology (placenta, umbilical cord). There was no significant between-group difference in the incidence of retained placenta. The maternal length of hospital stay after delivery in the dexamethasone group was slightly higher than in the matched group, but the difference was not statistically significant (p > 0.05). This could be explained by the higher rate of cesarean delivery in the dexamethasone group than in the matched group. In clinical practice at our study center, the maternal length of hospital stay after birth is usually 5 days for cesarean delivery and 3 days for normal delivery.

A previously published study evaluated the long-term impact of antenatal corticosteroids. In 2013, a follow-up study was conducted to evaluate the long-term behavioral, cognitive and developmental outcomes of children aged 8 – 15 years who were born preterm in a previous study with corticosteroid administration, and they did not exhibit any adverse events [27].

To the best of our knowledge, this is the first study of its kind carried out in Vietnam, where the National Standards still didn’t include the use of corticosteroids for late preterm labor between 34 0/7 weeks and 36 6/7 weeks of gestation. Further studies, especially randomized control trials could strengthen the evidence, and together with international professional guidelines and standards could help to update this management option for late preterm labor in Vietnam.

Limitations of this matched cohort study include the evaluation of only short-term complications of late preterm infants within 72 h after birth, and not the long-term complications. The sample size of this cohort study has been calculated by using the formula to estimate sample size for a proportion in observational study, and not the power in reducing proportion in a randomized control trial. The comparison of proportions and percentages of categories has been done by the whole group and not in pair-wise comparisons with Bonferroni correction. Another limitation is the nature of matched-cohort study, rather than randomized control trial, with a “best case scenario', which could not be similar to “effectiveness” in the real world setting.

## Conclusions

Our study showed that the administration of antenatal dexamethasone in the late preterm period could help to lower the proportion of respiratory distress in newborns. Dexamethasone administration significantly increased the proportion of neonatal hypoglycemia but not the percentages of other maternal or neonatal adverse outcomes.

## Supplementary Information

**Additional file 1.** Data collection tool.

## Data Availability

The datasets used and/or analyzed during the current study are available from the corresponding author on reasonable request.

## Abbreviations

ACS: Antenatal corticosteroids
LPI: Late preterm infant
NICU: Neonatal intensive care unit
RD: Respiratory distress
