# Supplementary material for: Antenatal dexamethasone use and respiratory distress in late preterm infants: results from first Vietnamese matched cohort study
Source: BMC Pregnancy Childbirth. 2021 Aug 7;21:546. doi: 10.1186/s12884-021-04019-6 (PMC8349074; doi:10.1186/s12884-021-04019-6)
Supplement: Supplementary file 1 — Additional file 1. Data collection tool. [file 12884_2021_4019_MOESM1_ESM.docx]

**DATA COLLECTION TOOL**

**“ANTENATAL DEXAMETHASONE USE AND RESPIRATORY DISTRESS**

**IN LATE PRETERM INFANTS: RESULTS FROM FIRST VIETNAMESE**

**MATCHED COHORT STUDY”**

*Questionnaire number: ………………….*

*Registration number:……………….*

| **No.** | **QUESTIONS** | **ANSWERS** |
| --- | --- | --- |
| **IDENTIFICATION** | | |
| Name of the mother: .…………………………………………………………………………….……. | | |
| Mobile phone number:……………………………… ………………………………………………… | | |
| **A** | **DEMOGRAPHIC CHARACTERISTICS** | |
| **A1** | Age: .……………………………………………………………………………………………. | |
| **A2** | Marital status | 1. Married 2. Single |
| **A3** | Occupation | 1. No employment 2. Employed  3. Self employment |
| **A4** | Date of admission: ………………………………………………………………….……….…. | |
| **A5** | Date of discharge: ……………………………………………………………………………… | |
| **B** | **OBSTETRICS CHARACTERISTICS** | |
| **B1** | Gravidity: ………………………………………………………………………………………. | |
| **B2** | History of preterm birth: ..……………………………………………………………………… | |
| **B3** | The last menstrual period: ......………………………………………………………………….. | |
| **B4** | Pregnancy due date: .....………………………………………………………………………… | |
| **B5** | Gestational age at admission:  ..…………………………………………………………………. | |
| **B6** | Were you given dexamethasone in this pregnancy at 34 - 36 weeks of gestation? (If Yes, answer B7-B8) | 1. Yes 2. No |
| **B7** | Dose of dexamethasone | 1. 6 mg 2. 12 mg  3. 18 mg 4. 24 mg |
| **B8** | Duration between first dose of dexamethasone and delivery:…………… | 1. 0 – 24h 2. 24 – 48h  3. 48 – 72h 4. $>$ 72h |
| **C** | **MATERNAL CHARACTERISTICS** | |
| **C1** | Date of delivery: ……………………………………………………………………………… | |
| **C2** | Method of delivery | 1. Vaginal delivery 2. Assisted delivery  3. Cesarean delivery (Forceps…) |
| **C3** | Cause of delivery | 1. Preterm labor (intact membranes)  2. Rupture of membranes  3. Preeclampsia  4. Previa placenta  5. Placental abruption  6. Fetal compromise  7. Elective (maternal medical conditions) |
| **C4** | Complications (If Yes, answer C4.1-C4.3) | 1. Yes 2. No |
| **C4.1** | Chorioamnionitis | 1. Yes 2. No |
| **C4.2** | Endometritis | 1. Yes 2. No |
| **C4.3** | Retained placenta | 1. Yes 2. No |
| **C5** | Length of hospital stay after delivery: ………………………………………………………… | |
| **D** | **NEONATAL CHARACTERISTICS** | |
| **D1** | Infant sex | 1. MALE 2. FEMALE |
| **D2** | Birth weight:………………………….………………………………………………………… | |
| **D3** | Gestational age at birth:…………….…...……………………………………………………… | |
| **D4** | Neonatal Resuscitation (D4.1 – D4.4) | |
| **D4.1** | Apgar score at 1 minute:……………….. | 1. $\geq$ 7 2. $<$ 7 |
| **D4.2** | Apgar score at 5 minutes:……………….. | 1. $\geq$ 7 2. $<$ 7 |
| **D4.3** | Need for resuscitation at birth | 1. Yes 2. No |
| **D5** | Respiratory outcomes (72 hours after birth) (D5.1 – D5.8) | |
| **D5.1** | Neonatal respiratory distress? | 1. Yes 2. No |
| **D5.2** | Classification of respiratory distress | 1. Mild 2. Moderate  3. Severe |
| **D5.3** | Respiratory distress syndrome? | 1. Yes 2. No |
| **D5.4** | Stage of respiratory distress syndrome | 1. Mild 2. Moderate  3. Severe |
| **D5.5** | Surfactant use? | 1. Yes 2. No |
| **D5.6** | Respiratory support: supplemental oxygen | 1. Yes 2. No |
| **D5.7** | Respiratory support: CPAP | 1. Yes 2. No |
| **D5.8** | Respiratory support: Mechanical ventilation | 1. Yes 2. No |
| **D6** | NICU admission | 1. Yes 2. No |
| **D7** | Length of hospital stay:………………………………………………………………………… | |
| **D8** | Other outcomes (first 28 days of life) (D8.1 – D8.7) | |
| **D8.1** | Early-onset neonatal sepsis | 1. Yes 2. No |
| **D8.2** | Jaundice requiring phototherapy | 1. Yes 2. No |
| **D8.3** | Neonatal hypoglycemia: ……………….  (If glucose level less than 2.2 mmol/L) | 1. Yes 2. No |
| **D8.4** | Intraventricular hemorrhage | 1. Yes 2. No |
| **D8.5** | Necrotizing enterocolitis | 1. Yes 2. No |
| **D8.6** | Stage of necrotizing enterocolitis  (modified Bell's Staging Criteria) | 1. I - suspected 2. II - definite  3. III - advanced |
| **D8.7** | Neonatal death | 1. Yes 2. No |

*Date____/____/20__*
